# The Role of Panx3 in Age-Associated and Injury-Induced Intervertebral Disc Degeneration

**DOI:** 10.3390/ijms22031080

**Published:** 2021-01-22

**Authors:** Meaghan Serjeant, Paxton M. Moon, Diana Quinonez, Silvia Penuela, Frank Beier, Cheryle A. Séguin

**Affiliations:** 1 Department of Physiology and Pharmacology, Schulich School of Medicine & Dentistry, The University of Western Ontario, London, ON N6A 5C1, Canada; meaghan.serjeant@gmail.com (M.S.); pmoon2019@meds.uwo.ca (P.M.M.); dquinon@uwo.ca (D.Q.); fbeier@uwo.ca (F.B.); 2 Bone and Joint Institute, The University of Western Ontario, London, ON N6A 5C1, Canada; silvia.penuela@schulich.uwo.ca; 3 Department of Anatomy and Cell Biology, Schulich School of Medicine & Dentistry, The University of Western Ontario, London, ON N6A 5C1, Canada

**Keywords:** pannexin 3, intervertebral disc degeneration, mechanical stress, needle puncture injury, transgenic mice

## Abstract

Pannexin 3 (Panx3) is a mechanosensitive, channel-forming glycoprotein implicated in the progression of post-traumatic osteoarthritis. Despite evidence for *Panx3* expression in the intervertebral disc (IVD), its function in this cartilaginous joint structure remained unknown. Using *Panx3* knockout mice, this study investigated the role of Panx3 in age-associated IVD degeneration and degeneration induced by annulus fibrosus (AF) needle puncture. Loss of Panx3 did not significantly impact the progression of age-associated histopathological IVD degeneration; however, loss of *Panx3* was associated with decreased gene expression of *Acan*, *Col1a1*, *Mmp13* and *Runx2* and altered localization of COLX in the IVD at 19 months-of-age. Following IVD injury in the caudal spine, histological analysis of wild-type mice revealed clusters of hypertrophic cells in the AF associated with increased pericellular proteoglycan accumulation, disruptions in lamellar organization and increased lamellar thickness. In *Panx3* knockout mice, hypertrophic AF cells were rarely detected and AF structure was largely preserved post-injury. Interestingly, uninjured IVDs adjacent to the site of injury more frequently showed evidence of early nucleus pulposus degeneration in *Panx3* knockout mice but remained healthy in wild-type mice. These findings suggest a role for Panx3 in mediating the adaptive cellular responses to altered mechanical stress in the IVD, which may buffer aberrant loads transferred to adjacent motion segments.

## 1. Introduction

As a leading cause of disability, back pain poses a significant socioeconomic burden that is only predicted to rise as the population ages [1,2,3]. While the causes of back pain are complex, several large-scale, cross-sectional studies have demonstrated a strong association between back pain and degeneration of the intervertebral discs (IVDs) [4,5,6]. Despite efforts to improve the management of back pain associated with IVD degeneration, no disease-modifying treatments currently exist.

The IVD is a fibrocartilaginous tissue that forms the joints of the vertebral column. Located between adjacent vertebrae, it functions to absorb axial loads and provide mobility to the spine. The IVD is a heterogeneous structure made up of three tissues: the central gelatinous nucleus pulposus (NP) contained by the concentric lamellar structure of the annulus fibrosus (AF), interposed between the cartilaginous endplates (CEPs) that anchor the IVDs to adjacent vertebrae. IVD function relies on the synergistic roles of its composite tissues. The hydrostatic properties of the NP, attributed to its high proteoglycan content, enable it to resist compressive loads [7]. The radial forces exerted by the NP are balanced by tensile loads generated across the collagen bundles of the AF lamellar network [7,8]. In addition to its structural role, the CEP enables nutrient and waste exchange between vertebral bodies and the largely avascular IVD [9].

Numerous risk factors, including genetics and age, can predispose the IVD to degeneration [7,10,11]. IVD degeneration propagates through a cascade of maladaptive cellular responses to biochemical or biomechanical changes in the microenvironment that lead to progressive structural deterioration and loss of tissue function [7,12]. Increased matrix degradation coupled with abnormal matrix synthesis in the NP contribute to reduced tissue hydration and impair the ability of the IVD to resist axial load [13,14]. Consequently, loads are transferred to the surrounding AF thereby altering its mechanical environment and contributing to gradual loss of AF tissue integrity, evidenced morphologically as lamellar disorganization and widened inter-lamellar spaces [7,8,15,16]. While it is clear that the mechanical environment is an important determinant of IVD health and disease, the molecular mechanisms that mediate responses of the IVD to mechanical stress are not fully understood.

Pannexin 3 (Panx3), one of three channel-forming proteins of the pannexin family, is abundantly expressed in skeletal tissues where it is implicated in processes of tissue development and disease [17,18,19,20]. Functionally, Panx3 channels are involved in the release of ATP at the cell surface and calcium from the endoplasmic reticulum, respectively, with channel activity detected following mechanical stimulation or membrane depolarization in vitro [17,18,21]. In cartilage, Panx3 is expressed in the prehypertrophic zone of the growth plate where it plays a role in reducing chondrocyte proliferation and promoting hypertrophic differentiation [17,19,20,22]. Previous work by our group investigating the role of Panx3 in post-traumatic osteoarthritis (OA) demonstrated that both cartilage-specific and whole-body *Panx3* knockout (*Panx3^-/-^*) mice were resistant to developing OA following destabilization of the medial meniscus [19]. Based on the these findings, we speculated that Panx3 could promote tissue breakdown in cartilage following joint destabilization by mediating hypertrophic differentiation of chondrocytes, a well-recognized driver of OA, highlighting a catabolic role for Panx3 in mechanically stressed articular cartilage [19,23].

Given the role of Panx3 in regulating physiological and pathological processes in cartilaginous tissues, we investigated *Panx3* expression in the IVD. Microarray analysis suggests a tissue-specific expression pattern of *Panx3* in the IVD, with approximately 30-fold greater expression detected in the AF relative to the NP in 2.5-month-old mice [24]. Despite evidence of *Panx3* expression in the IVD, its function in this tissue remains unknown. Using the established global *Panx3^-/-^* mouse model (germline gene deletion; [19]), the current study sought to investigate the hypothesis that Panx3 regulates the cellular responses to mechanical stress in the IVD. We investigate the role of Panx3 in the IVD using both age-associated and injury-induced models of IVD degeneration.

## 2. Results

### 2.1. Loss of Panx3 Does Not Alter Age-Associated IVD Degeneration

**Figure 1:**
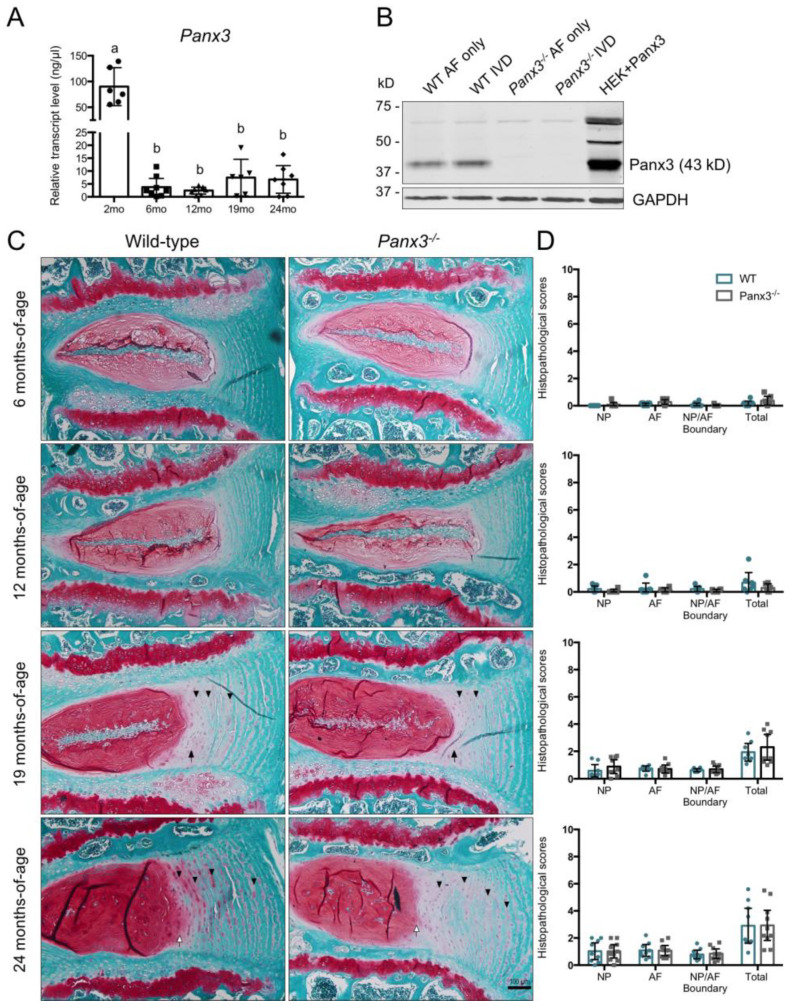
Loss of Panx3 does not alter age-associated intervertebral disc (IVD) degeneration. (**A**) Analysis of *Panx3* gene expression in thoracic IVDs from wild-type (WT) mice at 2, 6, 12, 19 or 24 months-of-age. Transcript levels were determined relative to a six-point standard curve and data are presented as mean ± 95% CI. Values corresponding to each experimental animal are indicated by individual data points on bar graph. Bars labelled with the same letter are not significantly different based on *p* < 0.05; one-way ANOVA followed by Tukey’s post-hoc test (n = 6–8 mice per group, 6–8 IVDs pooled per mouse). (**B**) Western blot analysis of Panx3 protein in whole IVD or annulus fibrosus (AF) tissue from 2-month-old WT and *Panx3^-/-^* mice. Panx3 overexpressing human embryonic kidney 293T cells (HEK + Panx3) served as positive control (expected size of Panx3 is 43kD). Images are representative of three biological replicates (8 IVDs from the lumbar and thoracic spine pooled per mouse). (**C**) Representative mid-sagittal sections of lumbar IVDs stained with safranin-O/fast green from 6, 12, 19 or 24-month-old WT and *Panx3^-/-^* mice. Black arrows indicate disruption in lamellar organization at the NP-AF boundary, white arrows indicate loss of defined NP-AF boundary, arrowheads indicate increased glycosaminoglycan (GAG) staining in the inter-lamellar matrix. Images are representative of 6–10 mice per group, 4–6 IVDs per mouse. (**D**) Histopathological scores comparing WT and *Panx3^-/-^* IVD tissues. Scores were assigned to the NP, AF and NP/AF boundary and summed for a total IVD score of 10. Higher scores represent a greater degree of degeneration. Data are presented as mean ± 95% CI (n = 6-10 mice per group, individual data points on bar graphs represent the average score of 4–6 IVDs per mouse).

We first sought to characterize *Panx3* expression in the IVD. Quantitative polymerase chain reaction (qPCR) analysis detected *Panx3* transcript levels in wild-type (WT) IVDs at all time points assessed, with significantly greater expression at 2 months-of-age compared to 6, 12, 19 and 24 months-of-age (Figure 1A). No significant differences in IVD *Panx3* expression were detected between the 6, 12, 19 or 24-month time points. Since microarray characterization suggested *Panx3* is preferentially expressed in the AF [24], we assessed Panx3 protein levels in intact IVD and AF tissues. Immunoblotting revealed robust Panx3 protein expression in both intact IVD and isolated AF samples, with no evidence of Panx3 protein in *Panx3^-/-^* mice (Figure 1B).

To investigate the role of Panx3 in age-associated IVD degeneration, WT and *Panx3^-/-^* mice were aged to 6, 12, 19 or 24 months and lumbar spines were harvested for histopathological analysis. Mid-sagittal sections stained with safranin-O/fast green revealed no overt differences in the histological appearance of lumbar IVDs between WT and *Panx3^-/-^* mice at any of the time points assessed (Figure 1C). At 6 and 12 months-of-age, IVDs from both WT and *Panx3^-/-^* mice appeared healthy with no histopathological features of degeneration in either the NP or AF (Figure 1C,D). IVD tissues from WT and *Panx3^-/-^* mice at 19 months-of-age showed early signs of IVD degeneration, including disruptions in the lamellar organization at the NP-AF boundary and glycosaminoglycan (GAG) accumulation in the inter-lamellar matrix of the inner AF (Figure 1C). At 24 months-of-age, IVD tissues from WT and *Panx3^-/-^* mice showed features of progressive degeneration including a loss of cellularity in the NP, loss of a defined NP-AF border, increased inter-lamellar space throughout the AF and lamellar reversal in the inner AF (Figure 1C). Histopathological scoring of degenerative changes showed no significant differences between IVD tissues of WT and *Panx3^-/-^* mice at any of the time points examined (Figure 1D).

To assess possible compensation between pannexin proteins in *Panx3^-/-^* mice, *Panx1* and full length *Panx2* gene expression were analyzed in IVDs from WT and *Panx3^-/-^* mice. No significant difference in *Panx1* gene expression was detected between WT and *Panx3^-/-^* IVDs at any of the time points examined (Supplementary Figure S1). *Panx2* expression was assessed but was not reliably detected in IVDs of WT or *Panx3^-/-^* mice.

### 2.2. Loss of Panx3 Is Associated with Altered Gene Expression in the IVD

**Figure 2:**
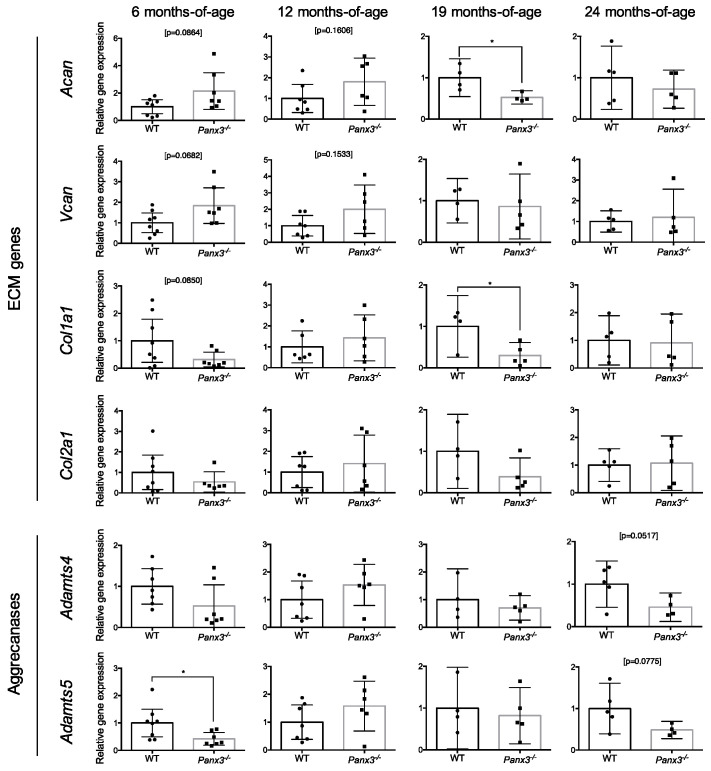
Effect of *Panx3* deletion on expression of extracellular matrix (ECM) and aggrecanase genes in the IVD. Analysis of *Acan*, *Vcan*, *Col1a1*, *Col2a1*, *Adamts4* and *Adamts5* gene expression in thoracic IVDs from 6, 12, 19 or 24-month-old WT and *Panx3^-/-^* mice. Gene expression was determined by relative quantification with values normalized to that of the *Rps29* housekeeper and expressed relative to age-matched WT controls. Data are presented as mean ± 95% CI (* indicates *p* < 0.05, Welch’s *t*-test; n = 4–8 mice per group, 6–8 IVDs pooled per mouse). Values corresponding to each experimental animal are indicated by individual data points on bar graphs.

To assess early changes in IVD health and degeneration, we quantified the expression of markers of IVD anabolism and catabolism. qPCR analysis of candidate genes demonstrated no significant differences in transcript levels of the extracellular matrix (ECM) genes *Acan*, *Vcan*, *Col1a1* or *Col2a1* between IVDs from WT and *Panx3^-/-^* mice at 6, 12 or 24 months-of-age (Figure 2). At 19 months-of-age, *Acan* and *Col1a1* expression were significantly decreased in IVDs from *Panx3^-/-^* mice relative to WT, while no significant differences in *Vcan* or *Col2a1* expression were detected between WT and *Panx3^-/-^* mice (Figure 2).

Next we investigated the expression of aggrecanases that contribute to ECM breakdown in IVD degeneration [25,26]. At 6 months-of-age, IVDs from *Panx3^-/-^* mice showed significantly reduced *Adamts5* expression compared to WT mice, while no difference in *Adamts4* expression was detected (Figure 2). No significant differences were detected in *Adamts4* or *Adamts5* expression between WT and *Panx3^-/-^* mice at 12, 19 or 24 months-of-age (Figure 2).

**Figure 3:**
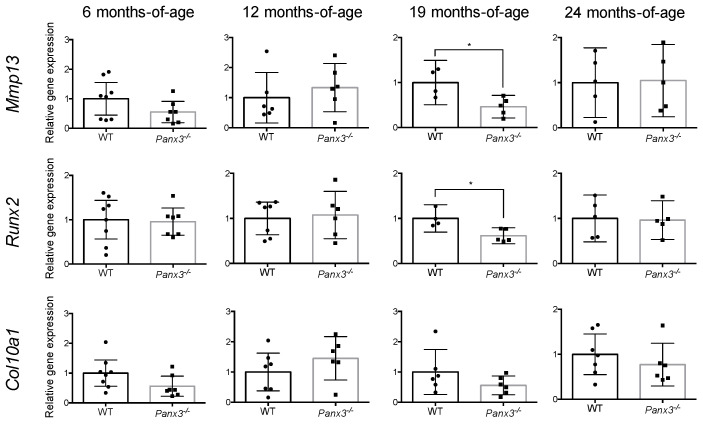
Effects of *Panx3* deletion on expression of hypertrophic chondrocyte genes in the IVD. qPCR analysis of *Mmp13*, *Runx2* and *Col10a1* gene expression in thoracic IVDs isolated from 6, 12, 19 or 24-month-old WT and *Panx3^-/-^* mice. Gene expression was determined by relative quantification with values normalized to that of the *Rps29* housekeeper and expressed relative to age-matched WT controls. Data are presented as mean ± 95% CI (* indicates *p* < 0.05, Welch’s *t*-test; n = 4–8 mice per group, 6–8 IVDs pooled per mouse). Values corresponding to each experimental animal are indicated by individual data points on bar graphs.

Given the role of Panx3 in promoting hypertrophic differentiation in chondrocytes [17], we assessed the expression of markers of chondrocyte hypertrophy in the IVD. At 6, 12 and 24 months-of-age, there were no significant differences in expression of *Mmp13*, *Runx2* or *Col10a1* in IVD tissues between age-matched WT and *Panx3^-/-^* mice (Figure 3). At 19 months-of-age, there was a significant decrease in the expression of *Mmp13* and *Runx2* in IVD tissues from *Panx3^-/-^* mice relative to age-matched WT but no differences in *Col10a1* expression (Figure 3).

### 2.3. Loss of Panx3 Alters the Localization of COLX in the IVD

**Figure 4:**
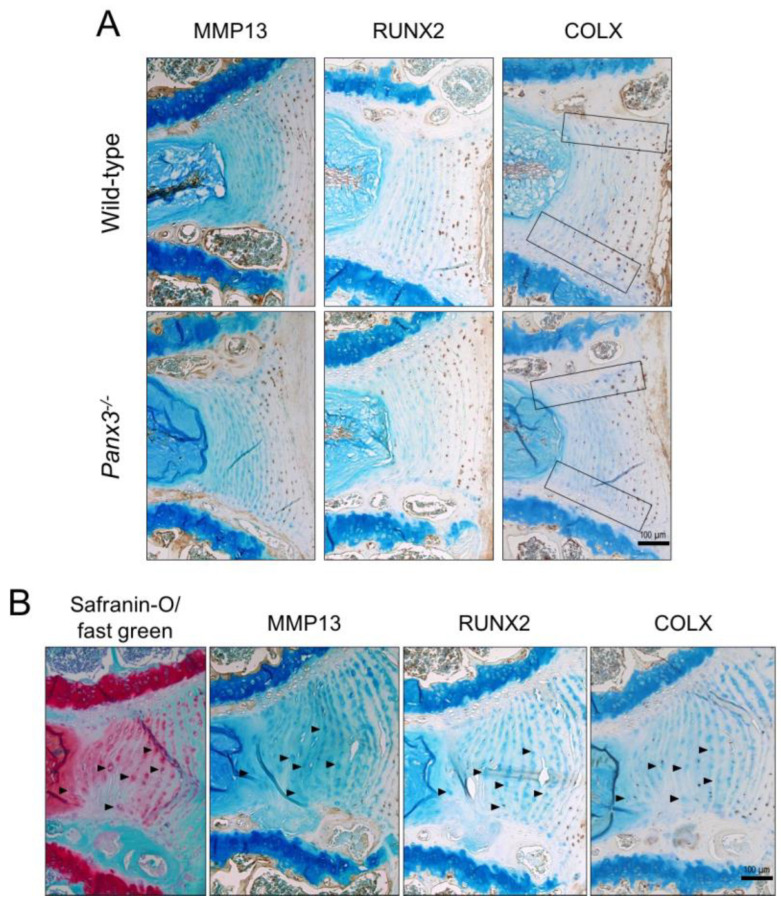
Localization of hypertrophic chondrocyte markers in *Panx3^-/-^* IVDs. (**A**) Representative mid-sagittal sections of lumbar IVDs from 19-month-old WT and *Panx3^-/-^* mice immunostained for either MMP13, RUNX2 or COLX (indicated by brown stain). Sections were counterstained with methyl green. Black boxes highlight the CEP-AF interface (n = 5 mice per group, 4–6 IVDs per mouse). (**B**) Serial sections of a representative WT lumbar IVD at 19 months-of-age stained with either safranin-O/fast green (red stain indicative of proteoglycan content) or anti- MMP13, RUNX2 or COLX antibody (indicated by brown stain) counterstained with methyl green. Arrowheads indicate enlarged AF cells.

To localize expression of hypertrophic markers within the IVD, we conducted immunohistochemistry in lumbar IVDs from WT and *Panx3^-/-^* mice at 19 months-of-age. Neither the abundance nor localization of MMP13 or RUNX2 in IVD tissues differed between WT and *Panx3^-/-^* mice. In addition to chondrocytes within the CEP and vertebral growth plates, MMP13 was primarily detected in the outer AF while RUNX2 was detected throughout the AF, with the highest density of staining localized to the outer AF (Figure 4A). In contrast, COLX staining was more abundant throughout the AF of WT mice compared to *Panx3^-/-^* mice, where staining appeared limited to the outer AF (Figure 4A). Additionally, COLX staining was more consistently detected across the AF-CEP boundary in WT compared to *Panx3^-/-^* mice where it was limited to the AF-CEP boundary in the outer AF (Figure 4A).

In keeping with previous reports [27,28], we noted the presence of enlarged hypertrophic cells in the AF at 19 and 24 months-of-age, coincident with the accumulation of degenerative changes. To determine if these cells phenotypically resemble hypertrophic chondrocyte, we assessed the localization of MMP13, RUNX2 and COLX. While enlarged AF cells were detected in both WT and *Panx3^-/-^* mice at 19 months-of-age, these hypertrophic cells did not consistently show expression of markers of chondrocyte hypertrophy (Figure 4B), suggesting a phenotype distinct from that of hypertrophic chondrocytes.

### 2.4. Loss of Panx3 Is Associated with Maintenance of AF Tissue Integrity Following IVD Injury

**Figure 5:**
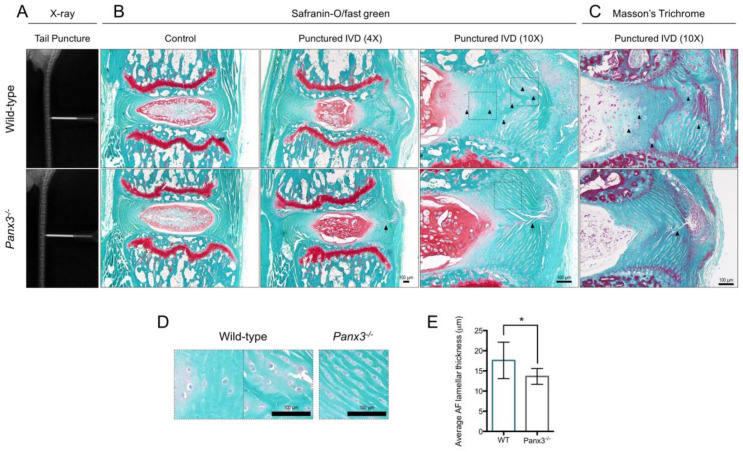
*Panx3^-/-^* mice maintain AF tissue architecture following NP depressurization of caudal IVDs. (**A**) Representative X-ray images of WT and *Panx3^-/-^* caudal IVDs undergoing needle puncture. (**B**,**C**) Representative mid-sagittal sections of caudal IVDs 7/8 and 8/9 from WT and *Panx3^-/-^* mice harvested 6-weeks following needle puncture stained with (**B**) safranin-O/fast green and (**C**) Masson’s Trichrome. Adjacent, uninjured caudal IVD 6/7 served as the control. Images representative of n = 6 mice per group, 2 IVDs per mouse. Arrowheads indicate enlarged AF cells detected in WT mice following injury, arrows mark the needle puncture track. (**D**) Magnified view of AF cells in WT and *Panx3^-/-^* caudal IVDs 6-weeks following needle puncture. Images correspond to areas indicated by boxes in panel B. (**E**) Average AF lamellar thickness in WT and *Panx3^-/-^* IVDs following needle puncture injury. Lamellar thickness (inclusive of lamellar and inter-lamellar widths) was measured throughout the AF of Mason’s Trichrome stained caudal IVDs post-puncture and averaged per IVD. (* indicates, *p* < 0.05, unpaired t-test; n = 6 mice per group, 2 IVDs per mouse).

Using a percutaneous IVD puncture model in the caudal spine (Figure 5A), we investigated the role of Panx3 in injury-induced IVD degeneration to assess its role in the context of altered disc biomechanics. In this model, acute injury is induced by a needle inserted through the AF into the central NP causing NP depressurization, mechanical instability and altered load distribution within the injured and adjacent IVDs [29,30]. Initial validation of this procedure confirmed the characteristic morphological changes in the IVD induced by AF injury, including loss of NP tissue evident immediately following puncture and fibrous NP repair tissue evident one week post-injury (Supplementary Figure S2E). Six weeks following injury, expected histopathological changes were detected in both WT and *Panx3^-/-^* mice, including loss of NP cell density and increased NP matrix accumulation compared to uninjured control IVDs (Figure 5B). While IVDs from both WT and *Panx3^-/-^* mice showed evidence of AF disruption associated with needle puncture, AF tissue architecture appeared better preserved in *Panx3^-/-^* IVDs compared to WT (Figure 5B). Specifically, hypertrophic cells were detected throughout the AF of WT mice following puncture but rarely detected in IVDs from *Panx3^-/-^* mice (Figure 5D). These enlarged AF cells were often found in clusters, appeared to contribute to widened inter-lamellar septa and were often associated with increased pericellular inter-lamellar GAG staining. Changes in ECM organization following puncture were assessed using Masson’s Trichrome staining (Figure 5C). In WT mice, the AF displayed disruptions in lamellar organization marked by areas of lamellar reversal, regions of undefined lamellar structure and loss of a defined NP-AF boundary. In contrast, the AF lamellar structure was generally preserved in *Panx3^-/-^* mice (Figure 5C). To quantify alterations in AF lamellar structure, we measured lamellar thickness (inclusive of both the lamellar and inter-lamellar widths), variables shown to increase with IVD age and degeneration [16,31,32], on the side of AF injury in WT and *Panx3^-/-^* mice. Following injury, a significant increase in the average lamellar thickness was detected in the AF of WT mice relative to *Panx3^-/-^* mice (Figure 5E).

### 2.5. Loss of Panx3 Accelerates NP Degeneration in IVDs Adjacent to Site of Puncture

**Figure 6:**
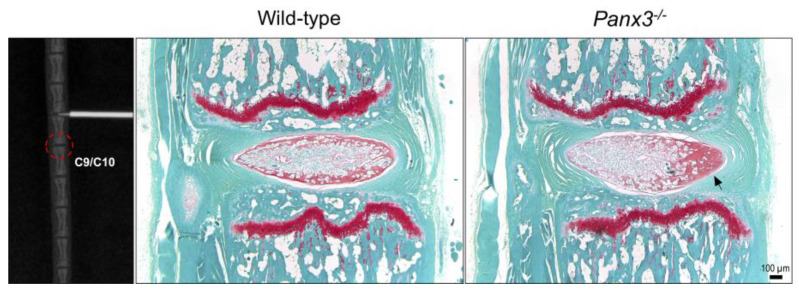
Degenerative changes identified in adjacent uninjured NP tissue of *Panx3^-/-^* mice following needle puncture. Representative X-ray image demonstrating caudal IVD 8/9 undergoing needle puncture injury. Dotted red circle highlights caudal IVD 9/10 of the motion segment distal to the site of injury. Representative safranin-O/fast green-stained mid-sagittal sections of uninjured caudal IVDs distal to the punctured IVDs, harvested 6-weeks after injury. Images are representative of n = 6 mice per group. Black arrow indicates accelerated NP degeneration marked by increased matrix density and reduced cellularity.

Although IVD injury was limited to caudal IVDs 7/8 and 8/9 in our model, we noted changes in histopathological features of the adjacent uninjured caudal IVD 9/10 in *Panx3^-/-^* mice. In WT mice, uninjured IVDs adjacent to the site of injury remained healthy 6 weeks post-injury (Figure 6). In contrast, in *Panx3^-/-^* mice, IVDs directly distal to the site of injury showed signs of accelerated NP degeneration, including increased matrix density and reduced cellularity on the side of injury (Figure 6); changes observed in 3/6 *Panx3^-/-^* mice and 0/6 WT mice.

## 3. Discussion

Panx3 is a channel-forming glycoprotein implicated in physiological and pathological functions in skeletal tissues. In cartilage, previous research suggests Panx3 mediates hypertrophic differentiation of growth plate chondrocytes [17,20,22] and the development of post-traumatic OA in articular cartilage [19]. Given the catabolic role of Panx3 in related tissues, we investigated the role of Panx3 in the IVD using previously characterized *Panx3^-/-^* mice [19]. Our findings show that while loss of Panx3 did not alter age-associated IVD degeneration, it appears to confer protection to the AF following acute NP herniation in an injury-induced model of IVD degeneration. Furthermore, our work suggests that Panx3 may play a role in the adaptive cellular response to altered mechanical load.

Consistent with previous transcriptomic analysis by our group [24], we confirmed robust expression of *Panx3* mRNA and protein in the IVD at 2 months-of-age. Our analysis also demonstrated a strong temporal regulation of *Panx3* expression in the IVD. Compared to expression levels at 2 months, expression of *Panx3* was decreased in the IVDs of mice from 6 to 24 months-of-age. IVDs of WT and *Panx3^-/-^* mice showed a similar progression of age-related degenerative changes as assessed by histopathological evaluation, with no evidence of compensatory upregulation in *Panx1* or *Panx2* gene expression in the IVD. ECM genes *Acan* and *Col1a1* and hypertrophic markers *Runx2* and *Mmp13*, were downregulated in *Panx3^-/-^* mice at 19 months-of-age, which may suggest subtle differences in the cellular microenvironment between WT and *Panx3^-/-^* mice. While decreased aggrecan in the NP is associated with degeneration [33,34], its increase in the AF is associate with early degenerative changes [33]. Similarly, while increased type I collagen is associated with NP fibrosis [34,35], it is the primary collagen of the AF lamellar network [36]. Since we assessed gene expression in intact IVDs, we cannot differentiate whether the observed decreases in *Acan* and *Col1a1* expression in *Panx3^-/-^* IVDs are indicative of a catabolic or anabolic response.

The downregulation in *Runx2* and *Mmp13* expression detected in *Panx3^-/-^* mice is intriguing given the role of Panx3 in promoting hypertrophic differentiation of chondrocytes and its reported role as a target of Runx2 [20]. Runx2 and Mmp13 are well-characterized in the context of chondrocyte hypertrophy and OA [23]. Elevated *Runx2* transcription was reported in human IVDs with moderate degeneration [37] and RUNX2 has been localized, along with other hypertrophic markers such as MMP13, to the NP and AF of degenerated human IVDs [25,38]. Previous work suggests a positive correlation between Panx3 and MMP13 expression in OA cartilage [19], consistent with the downregulated *Mmp13* expression we observed in IVDs of *Panx3^-/-^* mice. These data suggest a subtle protective role for the loss of *Panx3* in the IVD, associated with a delay in expression of hypertrophic markers at the onset of degeneration. Based on its role in articular cartilage, we considered that Panx3 may be regulating hypertrophic-like changes in the IVD. Despite the changes in gene expression, immunohistochemical analysis revealed no overt differences in MMP13 or RUNX2 localization between WT and *Panx3^-/-^* mice at 19 months-of-age, with subtle differences detected in COLX staining at the AF/CEP interface. Importantly, we demonstrate that enlarged cells detected in the AF with age-associated degeneration do not express classical markers of chondrocyte hypertrophy (i.e., MMP13, RUNX2, COLX). While the presence of enlarged AF cells has been reported as a histopathological feature of degeneration [27,28], to our knowledge the phenotype of these cells has not been directly investigated; our findings suggest that phenotypic changes associated with hypertrophy in the AF are distinct from those of chondrocytes and warrant further investigation.

Our finding of a subtle phenotype in *Panx3^-/-^* mice with age is consistent with previous characterization of pannexin knockout mouse models. Despite its broad expression, multiple *Panx1* knockout mouse models show seemingly normal anatomy and health [39,40]. In a *Panx3*-deficient mouse model, Yorgan et al., reported delayed ossification at birth but the absence of a skeletal phenotype in mature mice [41]. Similarly, characterization of the *Panx3^-/-^* mouse model used in this study demonstrated differences in the size of muscle attachment sites and diaphysis lengths [42] but overall normal skeletal development [19]. Of note, two additional *Panx3* knockout mouse models have been reported with more prominent bone abnormalities [22,43]. The reason for these phenotypic discrepancies may relate to differences in *Cre* drivers or background strains used to generate the transgenic mice [44]. Recent characterization of a *Panx1*/*Panx3* double knockout mouse model reported reduced body weight, decreased long bone length and alterations in skull shape and size in neonatal mice compared to WT, suggesting the importance of these proteins in early stages of skeletal development [45].

Interestingly, studies report more robust phenotypes in pannexin knockout mice in response to stress or injury [19,46,47]. Results from the current study using an injury-induced model of IVD degeneration support the hypothesis that pannexins contribute to tissue-specific adaptation to mechanical stress. Following injury, WT mice displayed characteristic changes in the AF including the accumulation of clusters of large, rounded cells associated with increased pericellular GAG staining and AF lamellar disorganization. In contrast, following NP depressurization, AF tissues in *Panx3^-/-^* mice maintained structural integrity and did not show evidence of cellular changes or matrix reorganization. These findings are in keeping with recent studies showing that *Panx3^-/-^* mice were resistant to surgically-induced OA [19]. Of interest, however, was the observation that IVDs adjacent to the site of injury (which experience altered biomechanics due to adjacent IVD depressurization) were prone to accelerated NP degeneration on the side of injury in *Panx3^-/-^* mice, changes not detected in WT mice. In humans, IVDs adjacent to immobilized motion segments following spinal fusion show accelerated degeneration as a consequence of altered mechanical stress [48]. Taken together, we speculate that changes in AF cell morphology and ECM deposition following injury in WT IVD tissues are evidence of an adaptive response to alterations in mechanical loading and contribute to the preservation of tissue homeostasis at adjacent IVD levels. The hypertrophic, GAG-producing AF cells at the site of injury may buffer mechanical stresses transferred to the AF by synthesizing an altered inter-lamellar ECM in response to the high compressive loads experienced following NP depressurization. This adaptive response may dampen transmission of aberrant loads to adjacent IVD tissues. Our data suggest a role for Panx3 in mediating the response of AF cells to altered mechanical stress, possibly through its functional role in ATP release at the cell surface or calcium release from the endoplasmic reticulum, as characterized in chondrocytes [17,18]. However, further investigation is required to better characterize the longitudinal response of *Panx3^-/-^* mice to IVD injury and to understand the underlying molecular mechanisms involved.

This work highlights a complex, context-dependent role of Panx3 in the IVD. Our analyses demonstrate that Panx3 does not play a significant role in the progression of age-associated IVD degeneration but may be involved in mediating the response to altered mechanical stress. We show that Panx3 is expressed in the AF where it may play a role in mediating responses to altered mechanical load, associated with the propagation of aberrant loads across spinal segments.

## 4. Materials and Methods

### 4.1. Experimental Animals

All experiments were performed in accordance with the policies and guidelines set forth by the Canadian Council on Animal Care and approved by the Animal Use Subcommittee of the University of Western Ontario (protocols 2017-154 and 2015-031). Genotyping confirmed homozygous deletion of *Panx3* in the whole-body *Panx3^-/-^* mice previously reported by our group [19]. Age-matched WT C57BL/6 mice were used as controls. Mice were housed in standard cages on a 12-h light/dark cycle with rodent chow and water available *ad libitum*. Mice were euthanized at 2, 3.5, 6, 12, 19 (±2 weeks) or 24 (±2 weeks) months-of-age. Intact lumbar (L1-L6) or caudal (C5-C12) spines were harvested for histological and immunohistochemical analyses. Thoracic IVDs (T5-T13; including NP, AF and CEP) were pooled (6–8 IVDs per mouse) for gene expression analyses. Intact IVDs or AF tissues were microdissected from alternating spinal levels (T3 to L6) and pooled (8 IVDs per mouse) for protein analysis.

### 4.2. Gene Expression Analysis

Immediately following dissection, thoracic IVDs from WT and *Panx3^-/-^* mice were placed in TRIzol (Life Technologies, Carlsbad, CA, USA), homogenized and total RNA extracted according to the manufacturer’s protocol. RNA concentrations were determined using a NanoDrop 2000 spectrophotometer (Thermo Scientific, Waltham, MA, USA), followed by reverse transcription of 350 ng of RNA/sample (iScript cDNA Synthesis Kit, Bio-Rad, Hercules, CA, USA). Gene expression was assessed by qPCR using the Bio-Rad CFX384 system (Hercules, CA, USA). Reactions were run in triplicate with 140 ng cDNA/reaction, with the exception of *Panx1*, *Panx2* and *Panx3* that were run with 420 ng cDNA/reaction. Each reaction contained 470 nM of forward and reverse primers (using previously validated primers and PCR parameters [47,49,50]; primer sequences provided in Supplementary Table S1) and 2 x SsoFast EvaGreen Supermix (Bio-Rad, Hercules, CA, USA). Transcript levels were quantified using ∆∆CT, normalized for input based on expression of *Rps29* [51,52] and expressed relative to age-matched WT controls. *Panx3* transcript levels were quantified relative to a six-point standard curve (1/5 serial dilution with initial input of 240 ng/µL) made from cDNA generated from IVD tissues at postnatal day 28.

### 4.3. Western Blot Analysis

Intact IVDs and AF tissues were harvested from 2-month-old (8 ± 2 weeks) WT and *Panx3^-/-^* mice for protein analysis. Human embryonic kidney 293T cells overexpressing mouse Panx3 (described in [21]) served as control. Total protein was harvested following tissue homogenization (PRO250 homogenizer, PRO Scientific, Oxford, CT, USA) and sonication (Sonic Dismembrator 100, Fisher Scientific, Waltham, MA, USA) in Triton-based extraction buffer as previously described [21]. Following quantification using the bicinchoninic acid assay, 16 μg total protein were separated by gel electrophoresis on a 10% sodium dodecyl sulfate-polyacrylamide gel and transferred to a nitrocellulose membrane. Membranes were blocked for 1.5 h with 3% (*w*/*v*) bovine serum albumin in phosphate buffer saline (PBS) and incubated overnight at 4 °C with rabbit polyclonal anti-Panx3 primary antibody (1:1000; described in [21]). Membranes were washed and incubated for 45 min with IRDye 800CW goat anti-rabbit secondary antibody (1:10,000; LiCor, Lincoln, NE, USA; 925-32211) prior to visualization using Odyssey LiCor infrared imaging system (Lincoln, NE, USA). GAPDH was detected using a mouse monoclonal primary antibody (1:5000; Millipore Sigma, Burlington, MA, USA; MAB374), followed by incubation with IRDye 680RD goat anti-mouse secondary antibody (1:10,000; LiCor, Lincoln, NE, USA; 925-68070).

### 4.4. Histology

Spines harvested for histological analyses were fixed overnight with 4% (*w*/*v*) paraformaldehyde in PBS, followed by 7 days of decalcification with Shandon’s TBD-2 (Thermo Scientific, Waltham, MA, USA). Following standard processing, tissues were embedded in paraffin and sectioned at a thickness of 5 μm. Mid-sagittal sections of lumbar and caudal spines were stained with 0.1% safranin-O/0.05% fast green or Masson’s Trichrome and imaged using a Leica DM1000 microscope (Wetzlar, Germany) with Leica Application Suite software. Safranin-O/fast green stained sections of lumbar spines were assessed for IVD degeneration based on an established histopathological scoring system [16] to assess the NP, AF and NP/AF boundary (4–6 IVDs scored per mouse). ImageJ (version 1.51s; National Institute of Health, Bethesda, MD, USA) was used to measure the lamellar widths in Masson’s Trichrome stained sections of caudal spines following AF puncture, defined as the distance between the medial edges of adjacent lamellae (inclusive of lamella and inter-lamellar matrix), perpendicular to the orientation of each lamella. Lamellar widths were assessed for all lamellae on the side of needle puncture with measurements taken as closed to the mid-IVD level as possible, while avoiding the needle puncture track. All lamellae on the side of puncture were measured and averaged for each IVD. A single section was assessed per IVD and two IVDs were assessed per mouse (n = 6 mice/group).

### 4.5. Immunohistochemical Analysis

Mid-sagittal sections of lumbar spines were used for immunohistochemical analyses. Antigen retrieval was performed in 0.1% (*v*/*v*) Triton-X followed by incubation in 3% (*v*/*v*) hydrogen peroxide in methanol. Slides were blocked in species-specific serum (5%) in PBS for 30 min at room temperature, followed by primary antibody incubation in a humidified chamber overnight at 4 °C. Primary antibodies were diluted in blocking solution as follows: rabbit polyclonal anti-COLX (1:750; abcam, Cambridge, England; ab58632); rabbit polyclonal anti-MMP13 (1:200; Proteintech, Rosemont, IL, USA; 18165-1-AP); rabbit polyclonal anti-RUNX2 (1:100; Novus Biologicals, Centennial, CO, USA; NBP1-77461). As negative control, slides were incubated overnight in blocking solution in the absence of primary antibody and IgG isotype controls were included for MMP13 and RUNX2 immunostaining. Slides were incubated in secondary antibody diluted in PBS [goat anti-rabbit for RUNX2 immunodetection (1:100; R&D Systems, Minneapolis, MN, USA; HAF008), goat anti-rabbit for MMP13 and COLX immunodetection (1:250; SantaCruz, Dallas, TX, USA; sc-2004)] for 1 h at room temperature. Secondary antibodies were conjugated with horseradish peroxidase and visualized following incubation with diaminobenzidine substrate (Dako Omnis, Santa Clara, CA, USA), followed by counterstaining with 0.5% methyl green.

### 4.6. Percutaneous IVD Needle Puncture

To induce IVD degeneration, we adapted a percutaneous needle injury model based on previous methods [53]. 2-month-old WT and *Panx3^-/-^* mice (n = 6 per genotype) were anesthetized using 1.75% isofluorane and dorsal view X-ray images were used to locate caudal IVDs in reference to a landmarking device (Supplementary Figure S2A). X-ray images were acquired at a peak energy of 60 kVp and a tube current of 20 mA using the PXM-20BT PLUS portable X-ray unit (United Radiology Systems Inc., Deerfield, IL, USA). Caudal IVDs 7/8 and 8/9 were marked on the dorsal side of the tail (Supplementary Figure S2B) then punctured with a 30-gauge needle inserted through the skin. The depth of puncture was standardized using a 22-gauge needle sleeve designed to expose 1.4 mm of the 30-gauge needle for a puncture depth at 50–70% of the IVD width (Supplementary Figure S2C). The needle was held in place for 45 s and depth of the puncture was confirmed with a lateral view X-ray (Supplementary Figure S2D). Following the procedure, mice were returned to conventional housing and euthanized 48 h, 1 week or 6 weeks later.

### 4.7. Statistical Analysis

Statistical analyses were performed using GraphPad Prism Software version 6.0c (San Diego, CA, USA). Data from qPCR analysis of *Panx3* gene expression was assessed using a one-way ANOVA followed by Tukey’s multiple comparison test. qPCR analyses comparing gene expression between *Panx3^-/-^* and WT mice were assessed using a two-tailed, unpaired t-test with Welch’s correction, followed by the ROUT outlier test. Histopathological scores were compared using a Mann-Whitney *U* nonparametric test. Average lamellar width measurements were compared using two-tailed, unpaired *t*-test. *p* < 0.05 was considered statistically significant.

## Supplementary Materials

Supplementary materials can be found at https://www.mdpi.com/1422-0067/22/3/1080/s1.

## Abbreviations

Acan	Aggrecan
ADAMTS-4	A disintegrin and metalloproteinase with thrombospondin motifs 4
ADAMTS-5	A disintegrin and metalloproteinase with thrombospondin motifs 5
AF	Annulus fibrosus
CEP	Cartilaginous endplates
CI	Confidence interval
Col1a1	Collagen, type I, alpha 1
Col10a1	Collagen, type X, alpha1
COLX	Collagen type X
Col2a1	Collagen, type II, alpha 1
ECM	Extracellular matrix
GAG	Glycosaminoglycan
IVD	Intervertebral disc
MMP-13	Matrix metalloproteinase-13
NP	Nucleus pulposus
OA	Osteoarthritis
Panx3	Pannexin 3
*Panx3^-/-^*	Whole-body *Panx3* knockout
PBS	Phosphate buffer saline
qPCR	quantitative polymerase chain reaction
Rps29	Ribosomal protein S29
Runx2	Runt-related transcription factor 2
Vcan	Versican
WT	Wild-type

## References

[1] Vos T., Abajobir A.A., Abbafati C., Abbas K.M., Abate K.H., Abd-Allah F., Abdulle A.M., Abebo T.A., Abera S.F., Aboyans V. (2017). Global, regional, and national incidence, prevalence, and years lived with disability for 328 diseases and injuries for 195 countries, 1990–2016: A systematic analysis for the Global Burden of Disease Study 2016. Lancet.

[2] Asche C.V., Kirkness C.S., McAdam-Marx C., Fritz J.M. (2007). The societal costs of low back pain: Data published between 2001 and 2007. J. Pain Palliat. Care Pharmacother..

[3] Hoy D., Bain C., Williams G., March L., Brooks P., Blyth F., Woolf A., Vos T., Buchbinder R. (2012). A systematic review of the global prevalence of low back pain. Arthritis Rheum..

[4] Cheung K.M.C., Karppinen J., Chan D., Ho D.W.H., Song Y.-Q., Sham P., Cheah K.S.E., Leong J.C.Y., Luk K.D.K. (2009). Prevalence and Pattern of Lumbar Magnetic Resonance Imaging Changes in a Population Study of One Thousand Forty-Three Individuals. Spine.

[5] Livshits G., Popham M., Malkin I., Sambrook P.N., MacGregor A.J., Spector T., Williams F.M.K. (2011). Lumbar disc degeneration and genetic factors are the main risk factors for low back pain in women: The UK Twin Spine Study. Ann. Rheum. Dis..

[6] Samartzis D., Karppinen J., Mok F., Fong D.Y.T., Luk K.D.K., Cheung K.M.C. (2011). A population-based study of juvenile disc degeneration and its association with overweight and obesity, low back pain, and diminished functional status. J. Bone Jt. Surg. Ser. A.

[7] Adams M.A., Roughley P.J. (2006). What is intervertebral disc degeneration, and what causes it?. Spine.

[8] Jensen G.M. (1980). Biomechanics of the lumbar intervertebral disc: A review. Phys. Ther..

[9] Urban J.P.G., Smith S., Fairbank J.C.T. (2004). Nutrition of the intervertebral disc. Spine.

[10] Battie M.C., Videman T., Gibbons L.E., Fisher L., Manninen H., Gill K. (1995). Determinants of lumbar disc degeneration: A study relating lifetime exposure and magnetic resonance imaging findings in identical twins. Spine.

[11] Buckwalter J.A. (1995). Aging and degeneration of the human intervertebral disc. Spine.

[12] Smith L.J., Nerurkar N.L., Choi K.-S., Harfe B.D., Elliott D.M. (2011). Degeneration and regeneration of the intervertebral disc: Lessons from development. Dis. Model. Mech..

[13] Boxberger J.I., Sen S., Yerramalli C.S., Elliott D.M. (2006). Nucleus pulposus glycosaminoglycan content is correlated with axial mechanics in rat lumbar motion segments. J. Orthop. Res..

[14] Le Maitre C.L., Pockert A.P., Buttle D.J., Freemont A.J., Hoyland J.A. (2007). Matrix synthesis and degradation in human intervertebral disc degeneration. Biochem. Soc. Trans..

[15] Adams M.A., McNally D.S., Dolan P. (1996). “Stress” distributions inside intervertebral discs. J. Bone Jt. Surg..

[16] Tam V., Chan W.C.W., Leung V.Y.L., Cheah K.S.E., Cheung K.M.C., Sakai D., Mccann M.R., Bedore J., Séguin C.A., Chan D. (2018). Histological and reference system for the analysis of mouse intervertebral disc. J. Orthop. Res..

[17] Iwamoto T., Nakamura T., Doyle A., Ishikawa M., De Vega S., Fukumoto S., Yamada Y. (2010). Pannexin 3 regulates intracellular ATP/cAMP levels and promotes chondrocyte differentiation. J. Biol. Chem..

[18] Ishikawa M., Iwamoto T., Nakamura T., Doyle A., Fukumoto S., Yamada Y. (2011). Pannexin 3 functions as an ER Ca 2+ channel, hemichannel, and gap junction to promote osteoblast differentiation. J. Cell Biol..

[19] Moon P.M., Penuela S., Barr K., Khan S., Pin C.L., Welch I., Attur M., Abramson S.B., Laird D.W., Beier F. (2015). Deletion of Panx3 prevents the development of surgically induced osteoarthritis. J. Mol. Med..

[20] Bond S.R., Lau A., Penuela S., Sampaio A.V., Underhill T.M., Laird D.W., Naus C.C. (2011). Pannexin 3 is a novel target for Runx2, expressed by osteoblasts and mature growth plate chondrocytes. J. Bone Miner. Res..

[21] Penuela S., Bhalla R., Gong X.-Q., Cowan K.N., Celetti S.J., Cowan B.J., Bai D., Shao Q., Laird D.W. (2007). Pannexin 1 and pannexin 3 are glycoproteins that exhibit many distinct characteristics from the connexin family of gap junction proteins. J. Cell Sci..

[22] Oh S.K., Shin J.O., Baek J.I., Lee J., Bae J.W., Ankamerddy H., Kim M.J., Huh T.L., Ryoo Z.Y., Kim U.K. (2015). Pannexin 3 is required for normal progression of skeletal development in vertebrates. FASEB J..

[23] Pitsillides A.A., Beier F. (2011). Cartilage biology in osteoarthritis—Lessons from developmental biology. Nat. Rev. Rheumatol..

[24] Veras M.A., McCann M.R., Tenn N.A., Séguin C.A. (2020). Transcriptional profiling of the murine intervertebral disc and age-associated changes in the nucleus pulposus. Connect. Tissue Res..

[25] Le Maitre C.L., Freemont A.J., Hoyland J.A. (2004). Localization of degradative enzymes and their inhibitors in the degenerate human intervertebral disc. J. Pathol..

[26] Pockert A.J., Richardson S.M., Le Maitre C.L., Lyon M., Deakin J.A., Buttle D.J., Freemont A.J., Hoyland J.A. (2009). Modified expression of the ADAMTS enzymes and tissue inhibitor of metalloproteinases 3 during human intervertebral disc degeneration. Arthritis Rheum..

[27] Tessier S., Tran V.A., Ottone O.K., Novais E.J., Doolittle A., DiMuzio M.J., Shapiro I.M., Risbud M.V. (2020). TonEBP-deficiency accelerates intervertebral disc degeneration underscored by matrix remodeling, cytoskeletal rearrangements, and changes in proinflammatory gene expression. Matrix Biol..

[28] Tolonen J., Gronblad M., Vanharanta H., Virri J., Guyer R., Rytomaa T., Karaharju E. (2006). Growth factor expression in degenerated intervertebral disc tissue—An immunohistochemical analysis of transforming growth factor beta, fibroblast growth factor and platelet-derived growth factor. Eur. Spine J..

[29] Yang F., Leung V.Y., Luk K.D., Chan D., Cheung K.M. (2009). Injury-induced sequential transformation of notochordal nucleus pulposus to chondrogenic and fibrocartilaginous phenotype in the mouse. J. Pathol..

[30] Martin J.T., Gorth D.J., Beattie E.E., Harfe B.D., Smith L.J., Elliott D.M. (2013). Needle puncture injury causes acute and long-term mechanical deficiency in a mouse model of intervertebral disc degeneration. J. Orthop. Res..

[31] Postacchini F., Bellocci M., Massobrio M. (1984). Morphologic changes in annulus fibrosus during aging. An ultrastructural study in rats. Spine.

[32] Marchand F., Ahmed A.M. (1990). Investigation of the laminate structure of lumbar disc anulus fibrosus. Spine.

[33] Cs-Szabo G., Juan D.R., Turumella V., Masuda K., Thonar E.J.A., An H.S. (2002). Changes in mRNA and protein levels of proteoglycans of the anulus fibrosus and nucleus pulposus during intervertebral disc degeneration. Spine.

[34] Antoniou J., Steffen T., Nelson F., Winterbottom N., Hollander A.P., Poole R.A., Aebi M., Alini M. (1996). The human lumbar intervertebral disc: Evidence for changes in the biosynthesis and denaturation of the extracellular matrix with growth, maturation, ageing, and degeneration. J. Clin. Investig..

[35] Tsai T.T., Lai P.L., Liao J.C., Fu T.S., Niu C.C., Chen L.H., Lee M.S., Chen W.J., Fang H.C., Ho N.Y.J. (2013). Increased periostin gene expression in degenerative intervertebral disc cells. Spine J..

[36] Eyre B.D.R., Muir H. (1976). Types I and II collagens in intervertebral disc. Biochem. J..

[37] Sato S., Kimura A., Ozdemir J., Asou Y., Miyazaki M., Jinno T., Ae K., Liu X., Osaki M., Takeuchi Y. (2008). The distinct role of the runx proteins in chondrocyte differentiation and intervertebral disc degeneration: Findings in murine models and in human disease. Arthritis Rheum..

[38] Rutges J.P.H.J., Duit R.A., Kummer J.A., Oner F.C., van Rijen M.H., Verbout A.J., Castelein R.M., Dhert W.J.A., Creemers L.B. (2010). Hypertrophic differentiation and calcification during intervertebral disc degeneration. Osteoarthr. Cartil..

[39] Bond S.R., Naus C.C. (2014). The pannexins: Past and present. Front. Physiol..

[40] Penuela S., Gehi R., Laird D.W. (2013). The biochemistry and function of pannexin channels. Biochim. Biophys. Acta.

[41] Yorgan T.A., Peters S., Amling M., Schinke T. (2019). Osteoblast-specific expression of Panx3 is dispensable for postnatal bone remodeling. Bone.

[42] Caskenette D., Penuela S., Lee V., Barr K., Beier F., Laird D.W., Willmore K.E. (2016). Global deletion of Panx3 produces multiple phenotypic effects in mouse humeri and femora. J. Anat..

[43] Ishikawa M., Williams G.L., Ikeuchi T., Sakai K., Fukumoto S., Yamada Y. (2016). Pannexin 3 and connexin 43 modulate skeletal development through their distinct functions and expression patterns. J. Cell Sci..

[44] Ishikawa M., Yamada Y. (2017). The role of Pannexin 3 in bone biology. J. Dent. Res..

[45] Abitbol J.M., O’Donnell B.L., Wakefield C.B., Jewlal E., Kelly J.J., Barr K., Willmore K.E., Allman B.L., Penuela S. (2019). Double deletion of Panx1 and Panx3 affects skin and bone but not hearing. J. Mol. Med..

[46] Penuela S., Kelly J.J., Churko J.M., Barr K.J., Berger A.C., Laird D.W. (2014). Panx1 regulates cellular properties of keratinocytes and dermal fibroblasts in skin development and wound healing. J. Investig. Dermatol..

[47] Abitbol J.M., Kelly J.J., Barr K., Schormans A.L., Laird D.W., Allman B.L. (2016). Differential effects of pannexins on noise-induced hearing loss. Biochem. J..

[48] Park P., Garton H.J., Gala V.C., Hoff J.T., McGillicuddy J.E. (2004). Adjacent segment disease after lumbar or lumbosacral fusion: Review of the literature. Spine.

[49] Ii H., Warraich S., Tenn N., Quinonez D., Holdsworth D.W., Hammond J.R., Dixon S.J., Séguin C.A. (2016). Disruption of biomineralization pathways in spinal tissues of a mouse model of diffuse idiopathic skeletal hyperostosis. Bone.

[50] McCann M.R., Patel P., Beaucage K.L., Xiao Y., Bacher C., Siqueira W.L., Holdsworth D.W., Dixon S.J., Séguin C.A. (2013). Acute vibration induces transient expression of anabolic genes in the Murine intervertebral disc. Arthritis Rheum..

[51] Kerr G.J., McCann M.R., Branch J.K., Ratneswaran A., Pest M.A., Holdsworth D.W., Beier F., Dixon S.J., Séguin C.A. (2017). C57BL/6 mice are resistant to joint degeneration induced by whole-body vibration. Osteoarthr. Cartil..

[52] Veras M.A., Tenn N.A., Kuljanin M., Lajoie G.A., Hammond J.R., Dixon S.J., Séguin C.A. (2019). Loss of ENT1 increases cell proliferation in the annulus fibrosus of the intervertebral disc. J. Cell. Physiol..

[53] Tian Z., Ma X., Yasen M., Mauck R.L., Qin L., Shofer F.S., Smith L.J., Pacifici M., Enomoto-Iwamoto M., Zhang Y. (2018). Intervertebral disc degeneration in a percutaneous mouse tail injury model. Am. J. Phys. Med. Rehabil..

