# Supplementary material for: The Role of Panx3 in Age-Associated and Injury-Induced Intervertebral Disc Degeneration
Source: Int J Mol Sci. 2021 Jan 22;22(3):1080. doi: 10.3390/ijms22031080 (PMC7865929; doi:10.3390/ijms22031080)
Supplement: Supplementary file 1 [file ijms-22-01080-s001.pdf]

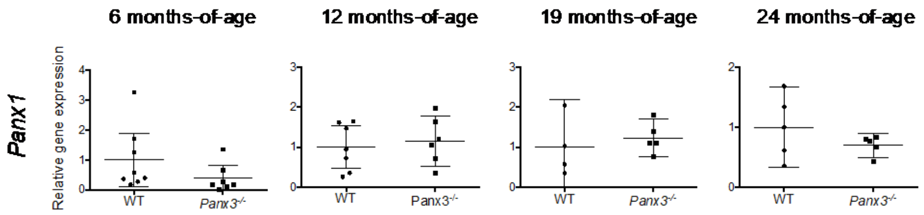

Supplementary Figure S1. *Panx1* expression is not altered in *Panx3*$^{-/-}$ mice. qPCR analysis of *Panx1* gene expression in thoracic IVDs of WT and *Panx3*$^{-/-}$ mice at 2, 6, 12, 19, or 24 months-of-age. Gene expression was determined by relative quantification with values normalized to that of the *Rps29* housekeeper and expressed relative to age-matched WT controls. Data are presented as mean ± 95% CI (n=4-8 mice per group. 6-8 IVDs pooled per mouse).

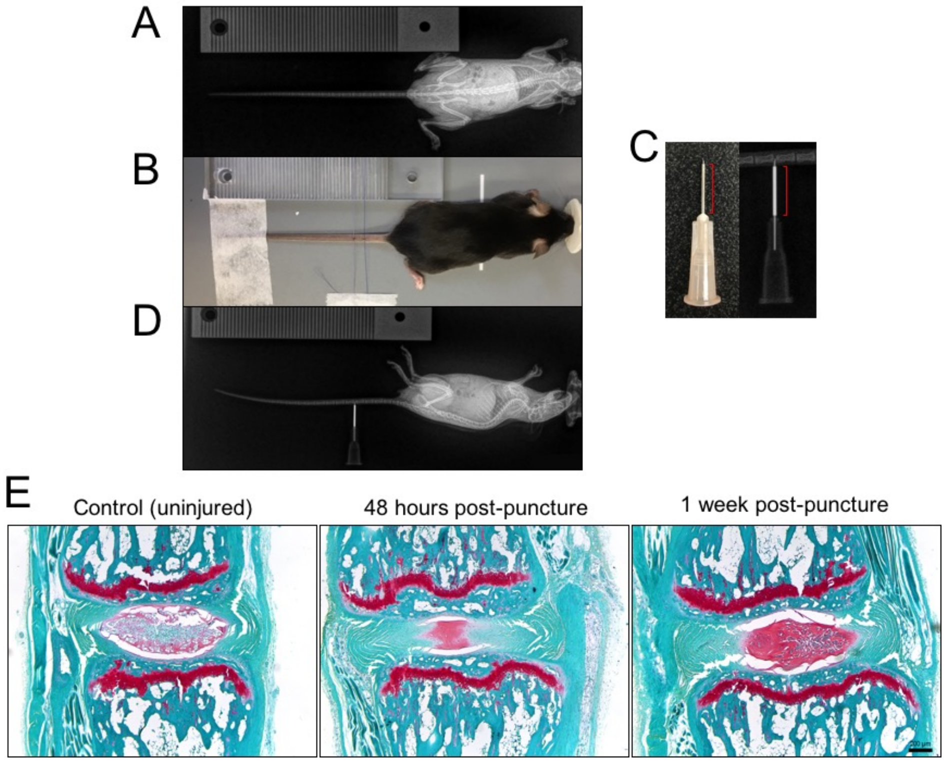

Supplementary Figure S2. Model of injury-induced IVD degeneration using percutaneous IVD needle puncture. Dorsal (A: X-ray; B: photograph) and sagittal (D: X-ray) view of a 2-month-old C57BL/6 mouse undergoing tail puncture procedure. (A) A dorsal X-ray is first taken to identify caudal IVDs 7/8 and 8/9 for puncture. (B) Using an electron dense measuring tool, the location of the IVDs are mapped out on the mouse tail. (C) A 30-gauge needle, guided by a 22-gauge sleeve (*red bracket*) to standardize depth of puncture, is then inserted through the skin into the IVD. (D) A sagittal X-ray is taken to confirm IVD puncture. (E) Mid-sagittal sections of control (non-punctured) and injured caudal IVDs stained with safranin-O/fast green demonstrate depressurization of the NP 48 hours following needle puncture, with some evidence of cell proliferation and formation of fibrous tissue repair detected after one week.

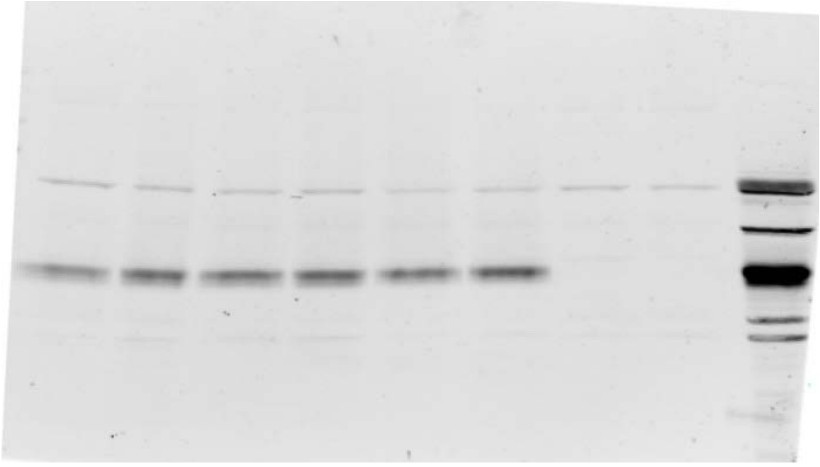

Supplementary Figure S3. Full unedited Western blot gel for Figure 1. Gel demonstrates PANX3 expression from whole IVD and AF tissues of three mice. From left to right, lanes represent WT 1 AF, WT 1 whole IVD, WT 2 AF, WT 2 whole IVD, WT 3 AF, WT 3 whole IVD, *Panx3*$^{-/-}$ AF (negative control), *Panx3*$^{-/-}$ whole IVD (negative control), Panx3 overexpressing human embryonic kidney (HEK) 293T cells (positive control). The five rightmost lanes are included in Figure 1.

Supplementary Table S1. qPCR Primer Sequences

Gene Symbol	Forward Primer (5′ to 3′)	Reverse Primer (5′ to 3′)
Acan	CTGGGATCTACCGCTGTGAAG	GTGTGGAAATAGCTCTGTAGTGGAA
Vcan	TTTTACCCGAGTTACCAGACTC	GGAGTAGTTGTTACATCCGTTG
Col1a1	CTGGCGGTTCAGGTCCAAT	TCCAGGCAATCCAGGAGC
Col2a1	GCACATCTGGTTTGGAGAGACC	TAGCGGTGTTGGGAGCCA
Mmp13	CTTCTTCTTGTTGAGCTGGAACTC	CTCTGTGGACCTCACTGTAGACT
Runx2	ATGGCTTGGGTTTCAGGTTAGGGA	TGGAGTGAAGGATGAGGGCAAACT
Adamts4	GAGGAGGAGATCGTGTTTCCAG	CAAACCCTCTACCTGCACCC
Adamts5	GGAGCGAGGCCATTTACAAC	GCGTAGACAAGGTAGCCCACTTT
Col10a1	GGGACCCCAAGGACCTAAAG	GCCCAACTAGACCTATCTCACCT
Panx1	ACAGGCTGCCTTTGTGGATTCA	GGGCAGGTACAGGAGTATG
Panx2	TGGTACCCATCCTGCTGGT	GGGTGAAGTTGTGCGGAGT
Panx3	TTTCGCCCAGGAGTTCTCATC	CCTGCCTGACACTGAAGTTG

Publisher's Note: MDPI stays neutral with regard to jurisdictional claims in published maps and institutional affiliations.

© 2021 by the authors. Submitted for possible open access publication under the terms and conditions of the Creative Commons Attribution (CC BY) license (http://creativecommons.org/licenses/by/4.0/).
